# The Conservation Status of the *Sphagnum* Species in Romania

**DOI:** 10.3390/plants15010140

**Published:** 2026-01-03

**Authors:** Miruna-Maria Ștefănuț, Sorin Ștefănuț, Ana-Maria Moroșanu

**Affiliations:** Institute of Biology Bucharest, Romanian Academy, 296 Splaiul Independenței, 060031 Bucharest, Romania; mirunastefanut@gmail.com (M.-M.Ș.); anamaria.morosanu@ibiol.ro (A.-M.M.)

**Keywords:** *Sphagnum*, threatened species, peatbogs, bryophytes, biodiversity conservation, Romania

## Abstract

The article presents the *Sphagnum* species of Romania and assesses their conservation status, based on the latest IUCN criteria. Based on the analyzed data, 35 *Sphagnum* species are confirmed for Romania (59.32% of *Sphagnum* Europe species) and 5 species are rejected. Over the past two years, three *Sphagnum* species have been confirmed as being new to Romania. The re-evaluation of the conservation status of *Sphagnum* species in Romania has led to the assessment of seven threatened species (20%), fewer than in the previous assessments (nine species). In the field, we have confirmed all 35 *Sphagnum* species of Romania. We present new records for *Sphagnum affine*, *S. balticum*, *S. cuspidatum*, *S. denticulatum*, *S. divinum*, *S. fimbriatum*, *S. inundatum*, *S. jensenii*, *S. majus*, *S. medium*, *S. obtusum*, *S. papillosum*, *S. platyphyllum*, *S. riparium*, *S. subfulvum*, *S. tenellum*, and *S. wulfianum*.

## 1. Introduction

The genus *Sphagnum* has attracted the attention not only of bryologists, but also of botanists, ecologists, conservationists, palynologists, and climate change researchers. *Sphagnum* L. species are included in the EU Habitats Directive, Annex V, except for *S. pylaesii* Brid., which is listed in Annex II (Directive 92/43/CEE) [1].

Comprising over 300 species worldwide, the genus *Sphagnum* represents one of the most significant moss groups in wetland ecosystems and is crucial to the creation and upkeep of peatbogs because it retains water and acidifies the environment, which prevents organic matter from decomposing and encourages the buildup of peat [2,3,4]. Peatlands store significant amounts of carbon (covering about 3% of the land surface), provide refuge for numerous rare species, and are considered priority ecosystems at the European level [5,6]. However, these habitats face significant threats from drainage, peat extraction, and climate change, all of which adversely impact biodiversity and the ecosystem services they provide. Consequently, as habitat builders, species of the genus *Sphagnum* have aroused major interest in attempts to combat the effects of climate change through large-scale restoration and reconstruction actions.

The Institute of Biology Bucharest has participated in the restoration of 23 peatbogs in the last ten years, an activity financed by four EEA grants. An additional 23 peatbogs have been restored in Romania by other institutions participating in the national peatbog restoration program, financed by the Ministry of Environment, Waters and Forests through the EEA—Environment Program.

The International Union for Conservation of Nature’s (IUCN) criteria, which incorporate variables such as population size, demographic trends, distribution region, and particular threats, are used to conduct the conservation assessments of *Sphagnum* species [7,8]. The re-evaluation of the conservation status of these species and their habitats has become crucial in recent years due to growing anthropogenic pressures, habitat degradation, and the availability of new dispersion data. This reassessment is critical to ensuring the long-term persistence of *Sphagnum* species within Romania and to inform efficient conservation strategies.

Bryological research in Romania began over 200 years ago, reflecting the long-standing scientific interest in the country`s bryophyte flora. The first book in which *Sphagnum* species were reported was published in 1846 [9], where Baumgarten J.C.G. cites five species: *Sphagnum capillifolium* (Ehrh.) Hedw., *S. compactum* Lam. & DC., *S. cuspidatum* Ehrh. ex Hoffm., *S. palustre* L., and *S. squarrosum* Crome. Since then, over 2000 articles on bryophyte species in Romania have been published, several hundred of which focus on *Sphagnum* species. The most comprehensive work appeared in 1998 and describes 32 *Sphagnum* species from Romania [10] (pp. 30–89).

Over time, 40 *Sphagnum* species have been published for Romania, of which 5 have been rejected. The conservation status of Romania’s bryophytes was assessed in 2012 [11], 2019 [7], and 2020 [12], reflecting changes in distribution patterns, habitat conditions, and emerging threats.

In Romania, *Sphagnum* species are dominant in the following habitats: active raised bogs (NATURA 2000 code: 7110), transition mires and quaking bogs (NATURA 2000 code: 7140), and bog woodland (NATURA 2000 code: 91D0); they are frequently found in depressions on peat substrates of the Rhynchosporion habitat (NATURA 2000 code: 7150); and they are pioneer species in degraded raised bogs still capable of natural regeneration (NATURA 2000 code: 7120) [1]. Additionally, *Sphagnum* species are frequently found in mountain forests, especially in spruce fir forests, in subalpine bushes, in subalpine meadows or on mountain wet rocks, up to 2500 m a.s.l. The *Sphagnum* species have been reported in blanket bogs (NATURA 2000 code: 7130) [1], but this type of habitat does not occur in Romania.

In this study, we present the updated list of *Sphagnum* species recorded in Romania, accompanied by a reassessment of their conservation status based on the latest IUCN criteria and recent field data, aiming to support ongoing conservation efforts.

## 2. Results

### 2.1. Sphagnum Species Rejected to Romania

Five *Sphagnum* species published for Romania have been rejected: *S. annulatum* H. Lindb. ex Warnst., *S. molle* Sull., *S. pulchrum* (Lindb. ex Braithw.) Warnst., *S. pylaesii* Brid. [10,13] and *S. magellanicum* Brid. [14].

*Sphagnum annulatum* was reported from Poiana Stampei Peatbog, Romania, 25 May 1925, *leg. & det.* Papp C., and Dofteana Valley, Târgu Ocna, Romania, 6 August 1951, *leg. & det*. Papp C. [15], *rev*. Plămadă E., as. *S. fallax* (H. Klinggr.) H. Klinggr. [10]; from Poiana Stampei Peatbog, 11.05.1957, *leg. & det*. Papp C. [15], *rev*. Plămadă E., as. *S. flexuosum Dozy & Molk*. [10]; and from Poiana Stampei Peatbog, 20 May 1957, 15 May 1959, *leg. & det*. Papp C. [15], *rev*. Plămadă E., as. *S. angustifolium* (C.E.O. Jensen ex Russow) C.E.O. Jensen [10]. In the BUCA—Romanian Academy Herbarium, we found *Sphagnum annulatum* samples from Poiana Stampei Peatbog, Romania, 25 July 1925, *leg. & det*. Papp C., and there are *S. angustifolium*, *rev.* Ștefănuț M.-M., 29.02.2025, BUCA *B6875*.

*Sphagnum molle* was reported from Poiana Stampei Peatbog, Romania, 23 July 1925, 20 May 1956, 15 May 1959, *leg. & det*. Papp C. [15], *rev*. Plămadă E., as. *S. fuscum* (Schimp.) H. Klinggr. [10]. However, we have not found samples of *S. molle* by Papp C. in the BUCA Herbarium.

**Figure 1:**
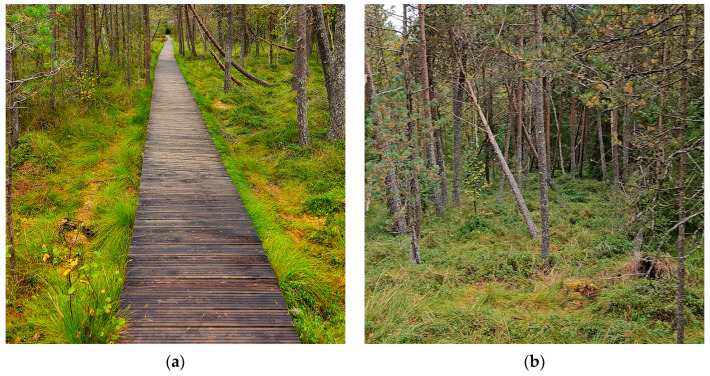
Poiana Stampei Peatbog, Romania, 19 September 2024: (**a**) a wooden bridge within the peatbog; (**b**) a view of the extensive *Sphagnum*-dominated area.

Between 2021 and 2024, we visited the Poiana Stampei Peatbog multiple times (Figure 1), but did not find the two species reported by Papp C., *Sphagnum annulatum* or *S. molle*.

*Sphagnum pulchrum* was reported from the Lăptici Peatbog, Bucegi Mountains, Southern Carpathians, Romania, 13 July 1940, *leg.* Cretzoiu P., *det.* Papp C., *rev*. Plămadă E., as *S. quinquefarium* (Braithw.) Warnst., FRE (Flora Romaniae Exssicata) *2393* [10]. The samples of *S. pulchrum* from the BUCA herbarium are all *S. quinquefarium*, *rev*. Ștefănuț M.-M., 20 May 2025, BUCA *B6982-B6985*.

**Figure 2:**
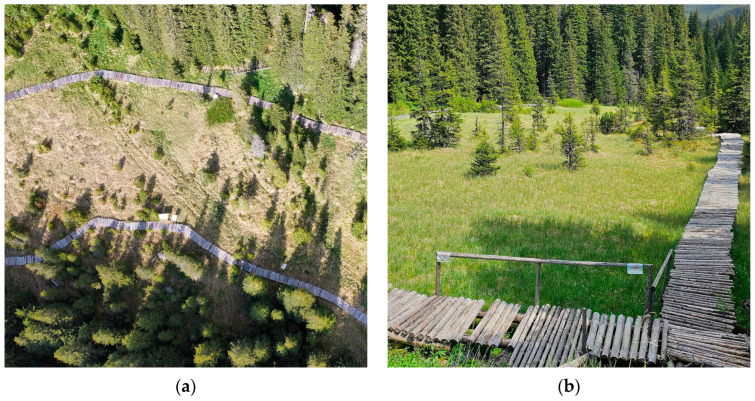
Lăptici Peatbog, Romania, 12 June 2025: (**a**) drone image of peatbog landscape; (**b**) *Sphagnum* area with the wooden bridge.

We visited the Lăptici Peatbog, Bucegi Mountains, in 2022 and 2025 (Figure 2), but we did not find *Sphagnum pulchrum*.

*Sphagnum pylaesii* was reported in 1972 by Debreczy Z. from the Retezat Mountains at Lia Lake, Ana Lake and Bucura Lake [16]. The samples were revised by Lange B. as *S. platyphyllum* (Lindb. ex Braithw.) Warnst. [17]. In 1999, Jakab G. reported *S. pylaesii* from the Tăul lui Dumitru Peatbog, Maramureș County, Romania, *leg*. Jakab G., *det*. E. Szurdoki [18], later revised by E. Szurdoki as *S. platyphyllum*.

**Figure 3:**
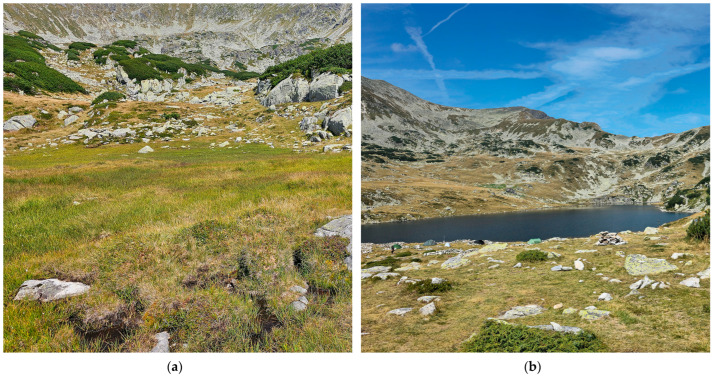
Retezat Mountains, Romania, 24 August 2024: (**a**) peatland on the edge of Lia Lake; (**b**) Bucura Lake with nearby peatland vegetation.

We visited the peatlands near Lia Lake and Bucura Lake, Retezat Mountains, in August 2024 (Figure 3), but we did not find *Sphagnum pylaesii*.

*Sphagnum magellanicum* was previously reported as a common species in Europe but it proved to be restricted only to South America; in Europe, only *S. medium* Limpr. and *S. divinum Flatberg & K*. Hassel are present [14].

### 2.2. Sphagnum Species Confirmed to Romania

**Figure 4:**
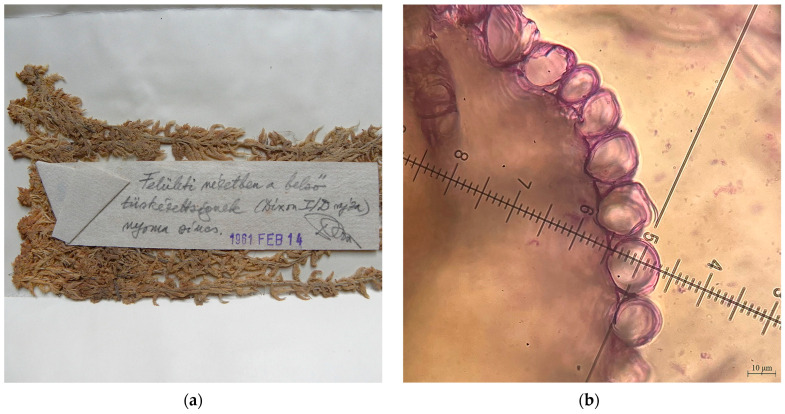
*Sphagnum affine* from Molhașul de la Râșca Peatbog, Cluj County, Romania, 25 October 1915, BP *7450/S*: (**a**) *S. affine*—herbarium samples; (**b**) *S. affine*—transversal section on branch leaf.

*Sphagnum affine Renauld & Cardot* was reported from the Molhașul de la Râșca Peatbog, Cluj County, Romania, 920–940 m a.s.l., 25 October 1915, *leg*. & *det*. Győrffy I., Péterfi M., as S. *imbricatum* Hornsch. var. *cristatum* Warnst. fo. *fuscescens* Warnst. BP (Budapest Herbarium) *7450/S*, *7451/S* [19], *rev*. Ștefănuț M.-M. [20] (Figure 4a,b). This was the first confirmed report of *S. affine* to Romania. *S*. *imbricatum* was reported also from the Mohoș Peatbog, Harghita County [21], but we did not find herbarium samples for verification. In the old reports of *S*. *imbricatum* in Europe were included *S. affine* and *S. austinii* Sull.

After almost 100 years, *S. affine* was found in the Molhașul de la Râșca Peatbog, 46.70849° N, 23.05224° E, 1070 m a.s.l., 26 July 2013, *leg*. Sass-Gyarmati A., Jakab G., *det.* Sass-Gyarmati A., 2014, EGR (Eger Herbarium), CL (Cluj-Napoca Herbarium), published on 30 June 2025 [22], BUCA *B12946*, *conf*. Ștefănuț M.-M., 23 June 2025.

**Figure 5:**
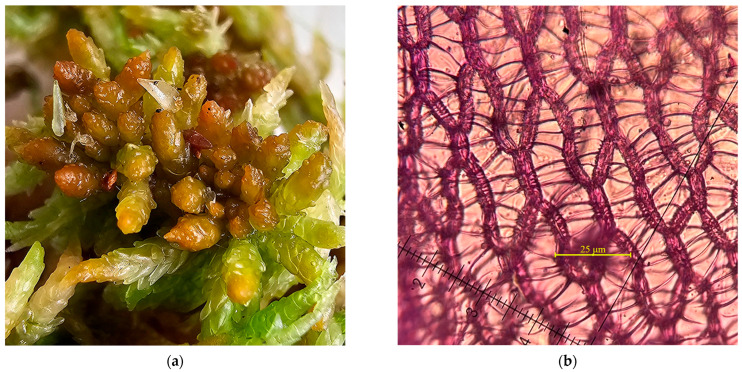
*Sphagnum affine* from Molhașul de la Râșca, Cluj County, Romania, 6 October 2025, BUCA *B12617*: (**a**) *S. affine*—close up; (**b**) *S. affine*—branch leaf with comb-lamellae.

Our study confirms the presence of *S. affine* at Molhașul de la Râșca—Dealul Negru Peatbog, Cluj County, 46.709174° N, 23.049022° E, 1053 m a.s.l., 6 October 2025, *leg.* Ștefănuț M.-M., Moroșanu A.-M., Ștefănuț S., *det.* Ștefănuț M.-M., *conf.* Ștefănuț S., BUCA *B12617* (Figure 5a,b).

**Figure 6:**
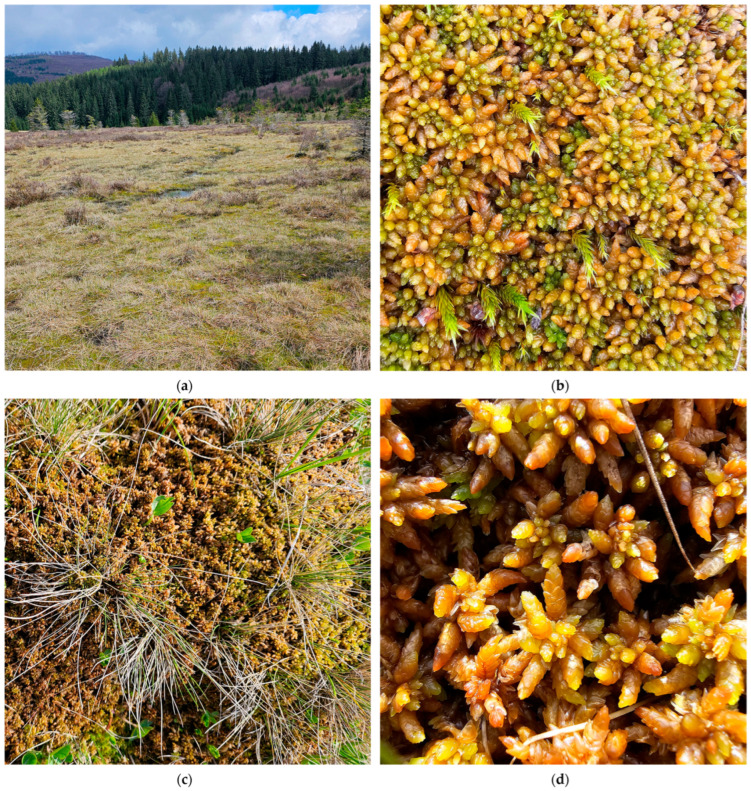
*Sphagnum papillosum* in Romania: (**a**) Vlășchinescu Peatbog—overview, 885 m a.s.l., 19 April 2024; (**b**) *S. papillosum*—close up, Vlășchinescu Peatbog, 19 April 2024; (**c**) low hummock with *S. papillosum* in Vâlcelul Clinului Bog, Bucegi Massif, 12 June 2025; (**d**) *S. papillosum*—close up, Vâlcelul Clinului Bog, Bucegi Massif, 12 June 2025.

*Sphagnum papillosum* Lindb. was recently reported from the Vlășchinescu Peatbog, Maramureș County, Romania, 885 m a.s.l., 19 April 2024, *leg.* & *det*. Ștefănuț M.-M., BUCA *B12295*, *B12311*, *B12312*, *B12325* (Figure 6a,b). Moreover, the species were reported from Molhașul de la Călățele, Cluj County, 916 m a.s.l., 25 October 1915, *conf*. Ștefănuț M-M., BP *7427*, Tăul Obcioarei Peatbog, Maramureș County, 1046–1047 m a.s.l., and Poiana Călineasa, Cluj County, 46.56278° N, 22.81636° E, 1367 m a.s.l., 1 October 2017, *leg*. & *det*. Hájková P. [20].

We found *S. papillosum* in Bucegi Massif, Vâlcelul Clinului Bog, in low hummocks, 45.356535° N, 25.463018° E, 1781 m a.s.l., 12 June 2025, *leg.* Moroșanu A.-M., Ștefănuț M.-M., Ștefănuț S., *det.* Ștefănuț M.-M., *conf*. Ștefănuț S., BUCA *B12525-B12529* (Figure 6c,d), alongside other bryophytes such as *S. capillifolium*, *S. compactum*, *S. palustre*, *S. rubellum* Wilson, *S. subnitens* Russow & Warnst., *Aulacomnium palustre* (Hedw.) Schwägr., and *Polytrichum strictum* Menzies ex Brid.

Additionally, we found *S. papillosum* in the Bihor Mountains, Tăul fără fund Peatbog, Padiș, Bihor County, 46.598770° N, 22.700088° E, 1231 m a.s.l., 9 October 2025, *leg*. Ștefănuț M.-M., Moroșanu A.-M., Ștefănuț S., *det.* Ștefănuț M.-M., *conf*. Ștefănuț S., BUCA *B12648*, *B12649*.

*Sphagnum jensenii* H. Lindb. was reported for the first time in 1959 in a small peatbog on Sărăcinul Mic Valley, Lotru Mountains, Southern Carpathians, 1260 m a.s.l., *leg.* Ștefureac T., Lungu L. & Popescu A., 1955–1956, *det.* Ștefureac T. [23]. Currently, the habitat is covered by water from the Vidra hydropower Lake. For this reason, we can say that this site of *S. jensenii* is extinct in Romania.

The second report of *S. jensenii* was in 1973 from Cristișor-Neagra Broștenilor Peatbog, Suceava County, *leg. & det.* Lungu L. [24].

Other *S. jensenii* reports have been included as synonyms of *S. annulatum*, which were later neither confirmed nor rejected.

**Figure 7:**
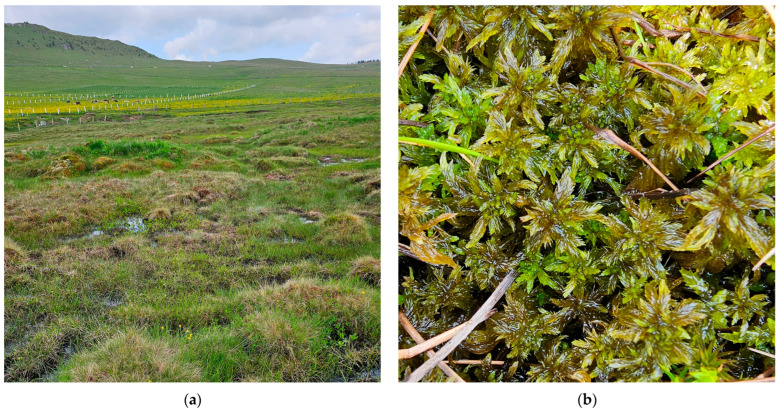
*Sphagnum jensenii* in the Vâlcelul Clinului Bog, Bucegi Massif, Romania, 12 June 2025: (**a**) Bucegi Massif, Vâlcelul Clinului Bog—overview, 1781 m a.s.l.; (**b**) *S. jensenii*—close up.

We confirmed the presence of the species *S. jensenii* in Romania from Bucegi Massif, Vâlcelul Clinului Bog, 45.356726° N, 25.462956° E, 1781 m a.s.l., 12 June 2025, *leg.* Ștefănuț M.-M., Ștefănuț S., *det.* Ștefănuț M.-M., *conf*. Ștefănuț S., BUCA *B12553* (Figure 7). This is the first report of *S. jensenii* in Romania in the last 50 years.

In Bucegi Massif, *S. jensenii* was found in the *Transition mires and quaking bogs* habitat (NATURA 2000 code: 7140), along with other bryophytes such as *Sphagnum angustifolium*, *S. contortum* Schultz, *S. fallax*, *S. girgensohnii* Russow, *S. inundatum* Russow, *S. majus* (Russow) C.E.O. Jensen subsp. *majus*, *S. platyphyllum*, *S. teres* (Schimp.) Ångstr., and *Hamatocaulis vernicosus* (Mitt.) Hedenäs. This bog was investigated by Ștefureac T. [25] and recently by Mountford J.O. and Onete M. [26].

**Figure 8:**
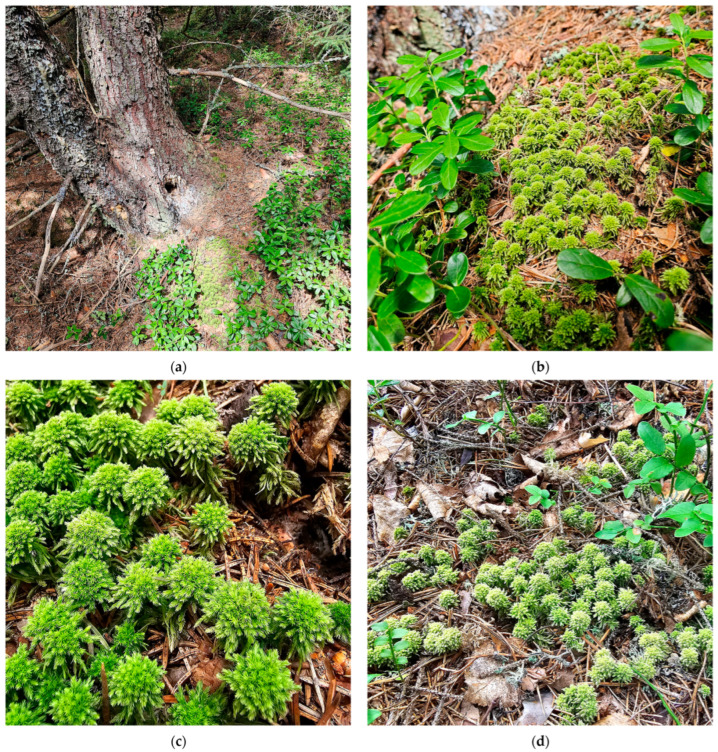
*Sphagnum wulfianum*, Apa Roșie, Covasna County, Romania, 20 August 2025: (**a**) Habitat of *S. wulfianum*, first record; (**b**) first record of *S. wulfianum* with *Vaccinium vitis-idaea*; (**c**) *S. wulfianum*, first record; (**d**) second record of *S. wulfianum* with *Vaccinium myrtifolium*.

*Sphagnum wulfianum* Girg. has not been found in the last 49 years, but recently, we rediscovered this species in Apa Roșie Peatbog, Covasna County, 20 August 2025, in two locations: 46.173667° N, 26.255000° E, 1002 m a.s.l., *leg.* Moroșanu A.-M., Ștefănuț M.-M., Ștefănuț S., *det.* Ștefănuț M.-M., *conf*. Ștefănuț S., BUCA *B12600* (Figure 8a–c), 46.174390° N, 26.255057° E, 1002 m a.s.l., *leg.* Ștefănuț M.-M., Moroșanu A.-M., Ștefănuț S., *det.* Ștefănuț M.-M., *conf*. Ștefănuț S., BUCA *B12601* (Figure 8d). *S. wulfianum* grows in forested peatland, with *S. capillifolium*, *S. divinum*, *S. girgensohnii*, and *S. medium*, on the bottom of *Picea abies* (L.) H. Karst., *Vaccinium vitis-idaea* L., and *V. myrtifolium* Michx.

*Sphagnum wulfianum* was reported for the first time in Romania in 1890 from Coșna Peatbog, Suceava County, and the Teșna Românești Peatbog, *leg*. Dörfler I., *det*. Breidler I. [27]. In 1925, this species was reported from the same site and from Poiana Stampei Peatbog, *leg.* & *det*. Mühldorf A. [28]. Moreover, *S. wulfianum* was reported from Drăgoiasa Peatbog, 19 August 1959, 12 August 1960, *leg.* & *det*. Ștefureac T., Suceava County [29], Valea Stânii Peatbog, Suceava County, 21 August 1969, *leg.* & *det*. Ștefureac T., Grădinița Peatbog, Suceava County, 25 August 1963, 9 August 1973, *leg.* & *det*. Ștefureac T. [30]. The last record of *S. wulfianum* in Romania is from the Apa Roșie Peatbog, Covasna County, 25 July 1975, *leg.* & *det*. Ștefureac T., Barabaș V., 15 July 1976, *leg.* & *det*. Ștefureac T. [31].

### 2.3. New Sphagnum Species Recently Published for Romania

**Figure 9:**
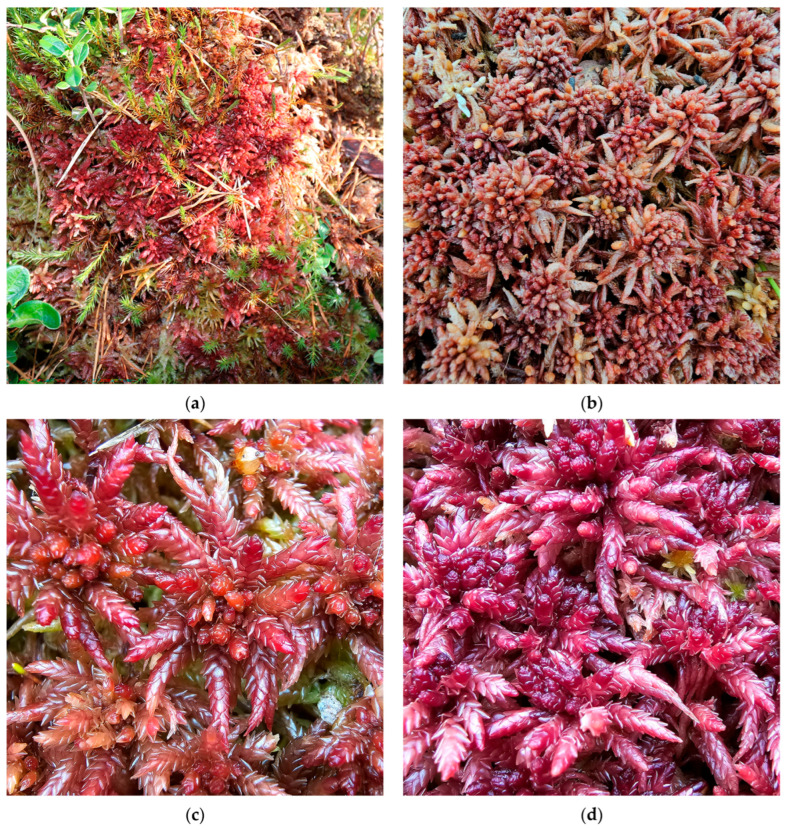
*Sphagnum divinum*, Romania: (**a**) *S. divinum*, Tinovul de lângă drum Peatbog, Lucina, Suceava County, 19 October 2023; (**b**) *S. divinum*, Mlaca Tătarilor Peatbog, Sibiu County, 12 March 2025; (**c**) *S. divinum*, Între Șimone Peatbog, Cluj County, 7 October 2025; (**d**) *S. divinum*, Tinovul de la Ic Ponor, Bihor County, 7 October 2025.

*Sphagnum divinum Flatberg & K*. Hassel was reported as new to Romania in 2024 from the Tinovul de lângă drum Peatbog, Lucina, Suceava County, 1159 m a.s.l., 19 October 2023, *leg.* & *det*. Ștefănuț M.-M., BUCA *B12274*, *B12275* [32] (Figure 9a).

This species was also found in other peatbogs: Tăul Obcioarei Peatbog, Maramureș Mountains, 47.684861° N, 24.536667° E, 1043 m a.s.l., 2014, *leg.* Goia I., *det.* Flatberg K.I., TRH (Trondheim Herbarium) *B96689/1*; Găina-Lucina Peatbogs, Suceava County, 47.647309° N, 25.195054° E, 1164 m a.s.l., 2014, *leg.* Goia I., *det.* Flatberg K.I., TRH *B96691/1*; Corund, Hargita County, 46.504176° N, 25.254747° E, 954 m a.s.l. 7 August 2024, *leg.* Nagy Z., *det.* Czołczyński B. [33]; Padiș-molidiș Peatbog, Molhașul Mare de la Izbuc Peatbog, Molhașul de la Călățele, Mluha Peatbog [22]; Avrig Peatbog, Avrig, Sibiu County, 405 m a.s.l. [34]; Pilugani Peatbog, Suceava County, 47.341472°N, 25.167588°E, 872 m a.s.l., 10 April 2024, *leg.* & *det*. Ștefănuț M.-M., BUCA *B12308* [35]; Iezerul Șureanu Peatbog, Șureanu Mountains, 45.580971° N, 23.508663° E, 1735 m a.s.l., 29 July 2021, *leg.* & *det*. Ștefănuț S., BUCA *B12331*; Șaru Dornei Peatbog, Suceava County, 47.258928° N, 25.356447° E, 902 m a.s.l., 4 September 2023, *leg.* & *det*. Ștefănuț M.-M., BUCA *B12294*; Teșna Românești Peatbog, Suceava County, 47.365580° N, 25.137234° E, 868 m a.s.l., 5 September 2023, *leg.* & *det*. Ștefănuț M.-M., BUCA *B12292*; Coșna Peatbogs, Suceava County [36], 47.374197° N, 25.167738° E, 858 m a.s.l., 20 October 2023, *leg.* & *det*. Ștefănuț M.-M., BUCA *B12282*, 19 September 2024, *leg.* Ștefănuț S., *det.* Ștefănuț M.-M., BUCA *B12326*, *B12327*; Hărnicești Peatbog, Maramureș County, 47.808603° N, 23.826399° E, 1007 m a.s.l., 21 April 2024, *leg.* & *det*. Ștefănuț M.-M., BUCA *B12289*, *B12390*, *B12391*; Peșteana Peatbog, Hunedoara County, 45.543508°N, 22.806266°E, 518 m a.s.l., 24 August 2024, *leg.* Ștefănuț S., *det.* Ștefănuț M.-M., BUCA *B12357*, *B12358*, *B12366*, *B12368- B12372*, 08 May 2025, *leg.* & *det*. Ștefănuț M.-M., BUCA *B12481*; Mlaca Tătarilor Peatbog, Sibiu County, 45.715082° N, 24.650163° E, 541 m a.s.l., 12 March 2025, *leg.* Moroșanu A.-M., Ștefănuț M.-M., *det.* Ștefănuț M.-M., BUCA *B12347* (Figure 9b); Tăul fără fund de la Poiana Mare-Obârșia Peatbog, Mehedinți County, 45.048267° N, 22.663262° E, 1035 m a.s.l., 06 May 2025, *leg.* Moroșanu A.-M., Ștefănuț M.-M., *det.* Ștefănuț M.-M., BUCA *B12483-B12486*; Bâlbâitoarea Peatbog, Prahova County, 45.389099° N, 26.104811° E, 863 m a.s.l., 26 June 2025, *leg.* Moroșanu A.-M., Ștefănuț S., *det.* Ștefănuț M.-M., BUCA *B12490*; Apa Roșie Peatbog, Covasna County, 46.174255° N, 26.250930° E, 1008 m a.s.l., 5 August 2025, *leg.* Ștefănuț M.-M., Moroșanu A.-M., Ștefănuț S., *det.* Ștefănuț M.-M., BUCA *B12576*; Apa Lină Peatbog, Covasna County, 46.151849° N, 26.203271° E, 972 m a.s.l., 6 August 2025, *leg.* Ștefănuț M.-M., Moroșanu A.-M., Ștefănuț S., *det.* Ștefănuț M.-M., BUCA *B12577- B12579*; Comandău Peatbog, Covasna County, 45.767559° N, 26.246558° E, 1020 m a.s.l., 7 August 2025, *leg.* Ștefănuț M.-M., Moroșanu A.-M., Ștefănuț S., *det.* Ștefănuț M.-M., BUCA *B12582*; Mohoș Peatbog, Harghita County, 46.135493° N, 25.900060° E, 1051 m a.s.l., 8 August 2025, *leg.* Ștefănuț M.-M., Moroșanu A.-M., Ștefănuț S., *det.* Ștefănuț M.-M., BUCA *B12584*; Între Șimone Peatbog, Cluj County, 46.645460° N, 22.846134° E, 1016 m a.s.l., 7 October 2025, *leg*. & *det*. Ștefănuț M.-M., *conf*. Ștefănuț S., BUCA *B12633* (Figure 9c); Tinovul de la Ic Ponor, Bihor County, 46.634990° N, 22.813090° E, 1037 m a.s.l., 7 October 2025, *leg*. & *det*. Ștefănuț M.-M., *conf*. Ștefănuț S., BUCA *B12632* (Figure 9d).

**Figure 10:**
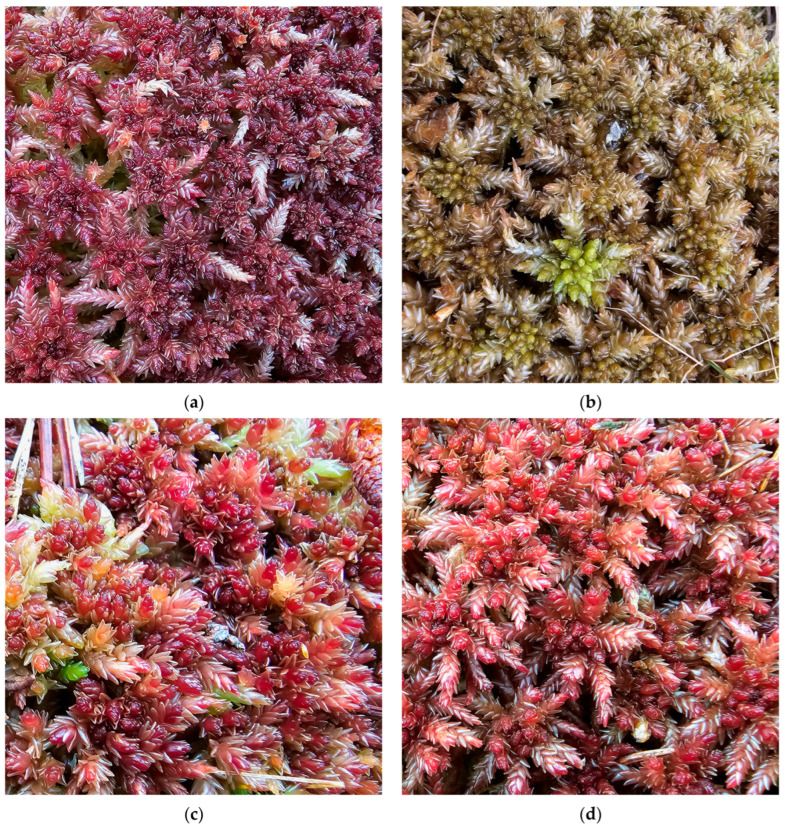
*Sphagnum medium*, Romania: (**a**) *S. medium* from the Avrig Peatbog, 12 March 2024; (**b**) *S. medium* from the Pilugani Peatbog, 10 April 2024; (**c**) *S. medium* from Molhașul Mare de la Izbuc, 8 October 2025; (**d**) *S. medium* from the Padiș Bălileasa Peatbog, 9 October 2025.

*Sphagnum medium* Limpr. was reported as new to Romania in 2024 from Avrig Peatbog, Avrig, Sibiu County, 405 m a.s.l., 12 March 2024, *leg.* & *det*. Ștefănuț M.-M., BUCA *B12280* (Figure 10a) [37]; Tinovul de lângă drum Peatbog, Lucina, Suceava County, 1160 m a.s.l., 19 October 2023, *leg.* & *det*. Ștefănuț M.-M., BUCA *B12283* [37]; Tăul de la Gutâi Peatbog, Gutâi Mountains, Maramureș County, 1057 m a.s.l., 9 October 2023, *leg.* Moroșanu A.-M., *det.* Ștefănuț M.-M., BUCA *B12293* [37]; Coșna Peatbogs, Suceava County [36]; Vlășchinescu Peatbog, Maramureș County, Romania, 885 m a.s.l., 19 April 2024, *leg.* & *det*. Ștefănuț M.-M. [20]; Molhașurile Căpățînii, Mluha Peatbog [22]; Pilugani Peatbog, Suceava County, 47.341555° N, 25.167579° E, 872 m a.s.l., 10 April 2024, *leg.* & *det*. Ștefănuț M.-M., BUCA *B12418*-*B12422* (Figure 10b) [35]; This species was also found in Mohoș Peatbog, Harghita County, 46.136389° N, 25.902778° E, 1051 m a.s.l., 2014, *leg.* Goia I., *det.* Flatberg K.I., TRH *B96689/1* [33]; Mlaca Tătarilor Peatbog, Sibiu County, 45.715082° N, 24.650163° E, 541 m a.s.l., 29 April 2011, *leg.* & *det*. Ștefănuț S., BUCA *B4364*; Tăurile Chendroaiei Peatbog, Gutâi Mountains, 47.709897° N, 23.835694° E, 1057 m a.s.l., 20 April 2024, *leg. & det.* M-M. Ștefănuț, BUCA *B12487*; Peșteana Peatbog, Hunedoara County, 45.543156° N, 22.806419° E, 517 m a.s.l., 24 August 2024, *leg.* Ștefănuț S., *det.* Ștefănuț M.-M., BUCA *B12373*- *B12375*, *B12417*, 8 May 2025, *leg.* Moroșanu A.-M., Ștefănuț M.-M., *det.* Ștefănuț M.-M., BUCA *B12488*; Bâlbâitoarea Peatbog, Prahova County, 45.389376° N, 26.105351° E, 863 m a.s.l., 26 June 2025, *leg.* Moroșanu A.-M., Ștefănuț S., *det.* Ștefănuț M.-M., BUCA *B12491*; Apa Roșie Peatbog, Covasna County, 46.176062° N, 26.251273° E, 1005 m a.s.l., 4 August 2025, *leg.* Ștefănuț M.-M., Moroșanu A.-M., Ștefănuț S., *det.* Ștefănuț M.-M., BUCA *B12575*; Apa Lină Peatbog, Covasna County, 46.151846° N, 26.203885° E, 972 m a.s.l., 6 August 2025, *leg.* Ștefănuț M.-M., Moroșanu A.-M., Ștefănuț S., *det.* Ștefănuț M.-M., BUCA *B12580*; Comandău Peatbog, Covasna County, 45.767594° N, 26.246503° E, 1021 m a.s.l., 7 August 2025, *leg.* Ștefănuț M.-M., Moroșanu A.-M., Ștefănuț S., *det.* Ștefănuț M.-M., BUCA *B12581*; Mohoș Peatbog, Harghita County, 46.135427° N, 25.899771° E, 1052 m a.s.l., 8 August 2025, *leg.* Ștefănuț M.-M., Moroșanu A.-M., Ștefănuț S., *det.* Ștefănuț M.-M., BUCA *B12585*; Între Șimone Peatbog, Cluj County, 46.645460° N, 22.846134° E, 1016 m a.s.l., 7 October 2025, *leg*. & *det*. Ștefănuț M.-M., *conf*. Ștefănuț S., BUCA *B12634*; Molhașul Mare de la Izbuc, Bihor County, 46.591455° N, 22.762728° E, 1209 m a.s.l., 8 October 2025, *leg*. & *det*. Ștefănuț M.-M., *conf*. Ștefănuț S., BUCA *B12636* (Figure 10c); Padiș Bălileasa Peatbog, Bihor County, 46.597889° N, 22.698577° E, 1233 m a.s.l., 9 October 2025, *leg*. & *det*. Ștefănuț M.-M., *conf*. Ștefănuț S., BUCA *B12637* (Figure 10d).

**Figure 11:**
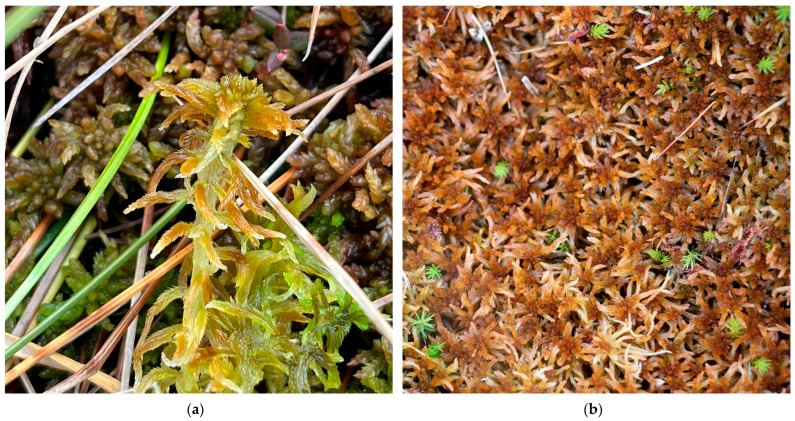
*Sphagnum subfulvum*, Romania: (**a**) *S. subfulvum* in the foreground and *S. medium* in the background, Tăurile Chendroaiei Peatbog, Gutâi Mountains, 20 April 2024; (**b**) *S. subfulvum*, Lacul Sec Peatbog, Siriu Mountains, 7 October 2021.

*Sphagnum subfulvum* Sjörs subsp. *subfulvum* was reported as new to Romania in 2025 from the Tăurile Chendroaiei Peatbog, Gutâi Mountains, Eastern Carpathians, 47.709897° N, 23.835694° E, 1057 m a.s.l., 20 April 2024, *leg. & det.* Ștefănuț M.-M., *conf*. Ștefănuț S. and Flatberg K.I., BUCA *B12320-B12323* [38], TRH *B124094/1* [33] (Figure 11a).

We also found *S. subfulvum* subsp. *subfulvum* in the Lacul Sec Peatbog, Siriu Mountains, Eastern Carpathians, 45.512310° N, 26.136964° E, 1464 m a.s.l., 7 October 2021, *leg*. Ștefănuț S.; *det.* Ștefănuț M.-M., *conf*. Ștefănuț S. and Flatberg K.I., BUCA *B12461* (Figure 11b), and in the Mluha Peatbog, Alba County, Detunatelor Mountains, Western Romanian Carpathians, 46.331161° N, 23.337354° E, 1334 m a.s.l., 3 November 2025, *leg*. Ștefănuț S., *det.* Ștefănuț M.-M., *conf*. Ștefănuț S., BUCA *B12659*.

### 2.4. New Records of Rare Sphagnum Species in Romania

Recently, new records of the rare *Sphagnum* species distribution in Romania have been reported.

**Figure 12:**
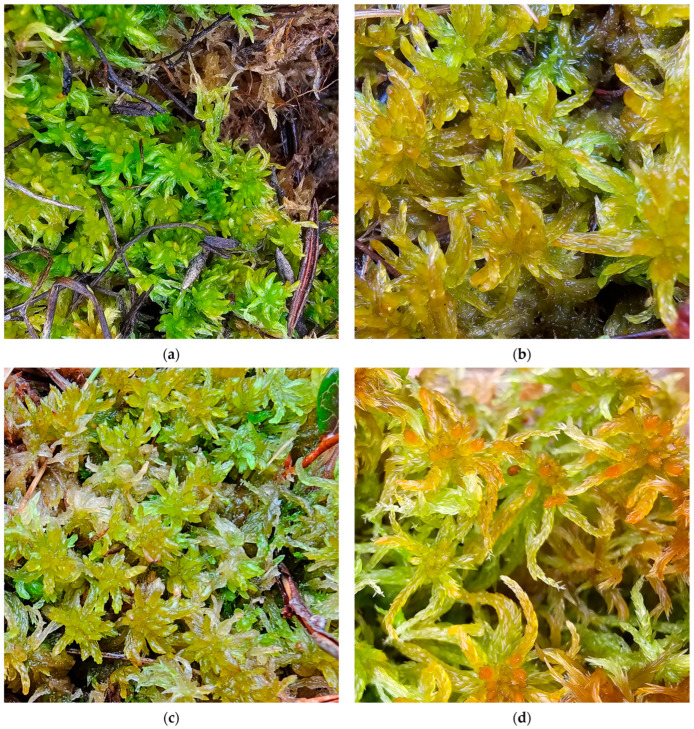
*Sphagnum tenellum*, Romania: (**a**) *S. tenellum*, Mohoș Peatbog, Harghita County, 8 August 2025; (**b**) *S. tenellum*, Molhașul Mare de la Izbuc Peatbog, Bihor County, 8 October 2025; (**c**) *S. tenellum*, Molhașurile din Valea Izbucelor Peatbogs, Bihor County, 8 October 2025; (**d**) *S. tenellum*, Tăul fără fund Peatbog, Bihor County, 9 October 2025.

*Sphagnum tenellum* (Brid.) Pers. ex Brid. was reported in Romania from Molhașul de la Călățele Peatbog, Cluj County, 6 May 1914, *leg*. & *det*. Peterfi M., TRH B109055/1 [33,39,40]; Bihor Mountains, Padiș, 21 July 1948, *leg*. Pop E. & Țopa E., *det*. Ștefureac T. [41]; Mehedinți County, Baia de Aramă region, 1947, *leg*. Țopa E., *det*. Ștefureac T. [41]; Bodoc Mountains, Zombor Valley, Băile Ceres [42]; Izbuc—Călineasa, 1180 m a.s.l., *leg*. Coldea G., *det*. Plămadă E. [10]. The species were recently confirmed from Molhașul de la Călățele, 46.72972° N, 23.02208° E, 920 m a.s.l., 24 July 2013, *leg*. & *det*. Sass-Gyarmati A. [22]. We found *S. tenellum* from Vlășchinescu Peatbog, Maramureș County, 47.7466° N, 23.7228° E, 885 m a.s.l., 19 April 2024, *leg*. & *det*. Ștefănuț M-M., *conf*. Ștefănuț S., Flatberg K.I., BUCA *B12382*, TRH *B:126517/1* [33]; Mlaștina fără fund de la Busești, Mehedinți County, 44.948156° N, 22.718931° E, 546 m a.s.l., 5 May 2025, *leg*. & *det*. Ștefănuț M-M., *conf*. Ștefănuț S. [43]; Mohoș Peatbog, Harghita County, 46.134799° N, 25.901594° E, 1049 m a.s.l., 8 August 2025, *leg*. & *det*. Ștefănuț M.-M., *conf*. Ștefănuț S., BUCA *B12593* (Figure 12a); Bihor Mountains, Molhașul Mare de la Izbuc, Bihor County, 46.591614° N, 22.762533° E, 1208 m a.s.l., 8 October 2025, *leg*. Ștefănuț M.-M., Moroșanu A.-M., Ștefănuț S., *det.* Ștefănuț M.-M., *conf*. Ștefănuț S., BUCA *B12639* (Figure 12b); Bihor Mountains, Molhașurile din Valea Izbucelor, Bihor County, 46.591163° N, 22.758000° E, 1210 m a.s.l., 8 October 2025, *leg*. Ștefănuț M.-M., Moroșanu A.-M., Ștefănuț S., *det.* Ștefănuț M.-M., *conf*. Ștefănuț S., BUCA *B12640*, *B12641* (Figure 12c); Bihor Mountains, Tăul fără fund Peatbog, Padiș, Bihor County, 46.598770° N, 22.700088° E, 1231 m a.s.l., 9 October 2025, *leg*. Ștefănuț M.-M., Moroșanu A.-M., Ștefănuț S., *det.* Ștefănuț M.-M., *conf*. Ștefănuț S., BUCA *B12621* (Figure 12d).

**Figure 13:**
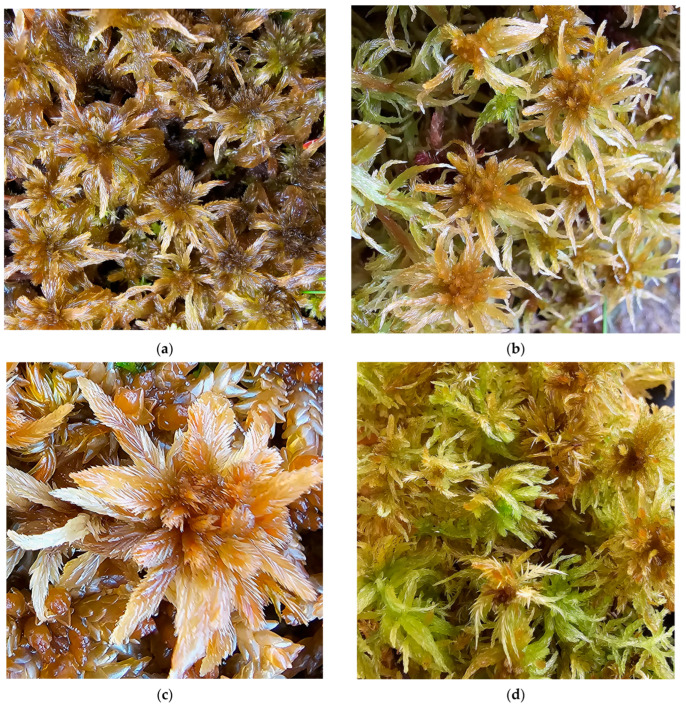
*Sphagnum balticum*, Romania: (**a**) *S. balticum*, Făgăraș Mountains, Valea Rea Peatbogs, 11 September 2025; (**b**) *S. balticum*, Molhașul Mare de la Izbuc, Bihor County, 9 October 2025; (**c**) *S. balticum*, Tăul fără fund Peatbog, Padiș, 9 October 2025; (**d**) *S. balticum*, Mluha Peatbog, Alba County, 3 November 2025.

*Sphagnum balticum* (Russow) C.E.O. Jensen has been reported from Mlaștina fără fund de la Busești, Mehedinți County, 44.948156° N, 22.718931° E, 546 m a.s.l., 5 May 2025, *leg*. & *det*. Ștefănuț M-M., *conf*. Ștefănuț S., Flatberg K.I., BUCA *B12518* [43]; Făgăraș Mountains, Valea Rea subalpine bogs, 45.598493° N, 24.755599° E, 2024 m a.s.l., 11 September 2025, *leg*. Ștefănuț S., *det.* Ștefănuț M.-M., *conf*. Ștefănuț S., BUCA *B12615* (Figure 13a); Bihor Mountains, Molhașul Mare de la Izbuc, Bihor County, 46.591614° N, 22.762533° E, 1208 m a.s.l., 8 October 2025, *leg*. Ștefănuț M.-M., Moroșanu A.-M., Ștefănuț S., *det.* Ștefănuț M.-M., *conf*. Ștefănuț S., BUCA *B12643* (Figure 13b); Bihor Mountains, Tăul fără fund, Padiș, Bihor County, 46.598770° N, 22.700088° E, 1231 m a.s.l., 9 October 2025, *leg*. Ștefănuț M.-M., Moroșanu A.-M., Ștefănuț S., *det.* Ștefănuț M.-M., *conf*. Ștefănuț S., BUCA *B12642* (Figure 13c); Mluha Peatbog, Alba County, Detunatelor Mountains, Western Romanian Carpathians, 46.331137° N, 23.337334°E, 1334 m a.s.l., 3 November 2025, *leg*. Ștefănuț S., *det.* Ștefănuț M.-M., *conf*. Ștefănuț S., BUCA *B12656-B12658* (Figure 13d).

**Figure 14:**
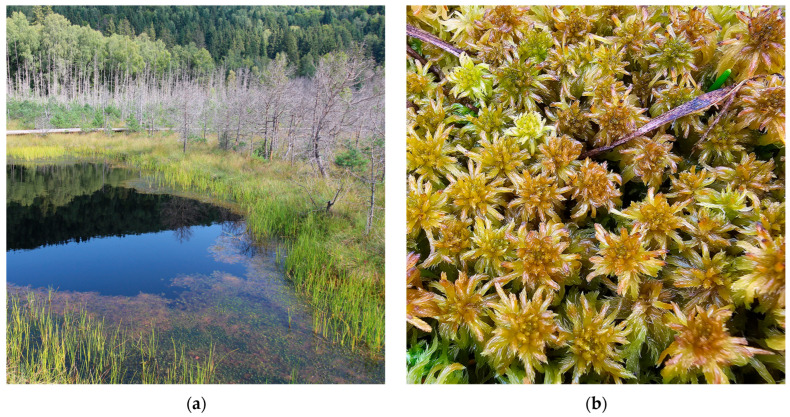
*Sphagnum obtusum* in the Mohoș Peatbog, Harghita County, Romania, 8 August 2025: (**a**) Mohoș Peatbog—overview; (**b**) *S. obtusum*—close up.

*Sphagnum obtusum* Warnst. was reported in the last ten years from Obârşia Lotrului, Vâlcea County, 45.43292° N, 23.66372° E, 1305 m a.s.l., 4 August 2018, *leg*. Hájková P. & Hájek M. 2018/128, *det.* Hájková P., BRNU (Brno Herbarium) *680362* [33]. We found *S. obtusum* in the Mohoș Peatbog, Harghita County, in hollows, 46.134819° N, 25.901645° E, 1048 m a.s.l., 8 August 2025, *leg*. & *det*. Ștefănuț M.-M., *conf*. Ștefănuț S., BUCA *B12583* (Figure 14).

**Figure 15:**
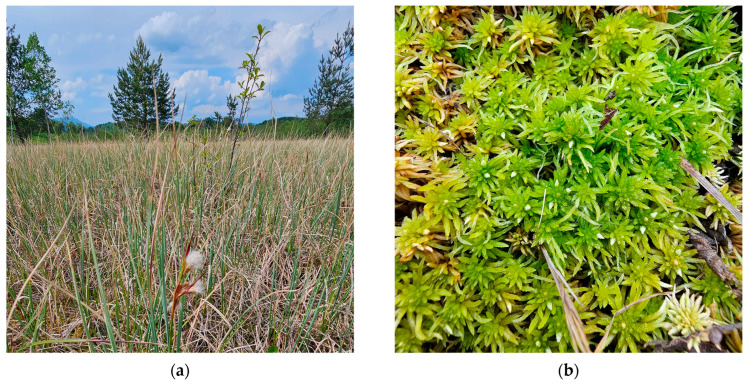
*Sphagnum fimbriatum*, Mlaștina fără fund de la Busești, Mehedinți County, Romania, 5 May 2025: (**a**) Busești Peatbog—overview; (**b**) *S. fimbriatum*—close up.

We found *Sphagnum fimbriatum* Wilson in Mlaștina fără fund de la Busești, Mehedinți County, 44.948896° N, 22.718289° E, 546 m a.s.l., 5 May 2025, *leg*. & *det*. Ștefănuț M-M., *conf*. Ștefănuț S., BUCA *B12510* (Figure 15). This is the first record for the Oltenia Region of Romania [10].

**Figure 16:**
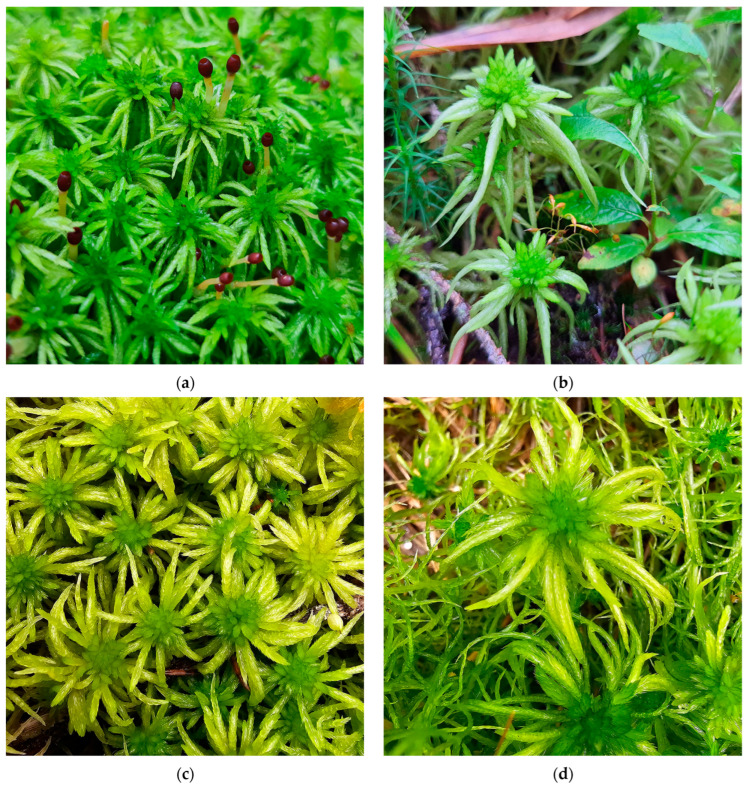
*Sphagnum riparium*, Romania: (**a**) *S. riparium* with sporophytes, Poiana Stampei Peatbog, Suceava County, 31 August 2023; (**b**) *S. riparium* and *Trematodon ambiguus* (Hedw.) Hornsch., Poiana Stampei Peatbog, Suceava County, 31 August 2023; (**c**) *S. riparium*, Între Șimone Peatbog, Cluj County, 7 October 2025; (**d**) *S. riparium*, Poiana Vărășoaia Peatbog, Bihor County, 9 October 2025.

*Sphagnum riparium* Ångstr. were found in Poiana Stampei Peatbog, Suceava County, 47.2955° N, 25.118722° E, 923 m a.s.l., 31 August 2023, 19 September 2024, *leg*. & *det*. Ștefănuț M.-M., *conf*. Ștefănuț S., BUCA *B12302*, *B12303* (Figure 16a,b). This is the first confirmed report of *S. riparium* for the Poiana Stampei Peatbog after 50 years [10,15]. Additionally, we found *S. riparium* in the Între Șimone Peatbog, Cluj County, 46.644809° N, 22.844885° E, 1012 m a.s.l., 7 October 2025, *leg*. Ștefănuț M.-M., Ștefănuț S., *det.* Ștefănuț M.-M., *conf*. Ștefănuț S., BUCA *B12627*, *B12628* (Figure 16c); Molhașul din Groapă Peatbog, Bihor County, 46.593992° N, 22.761243° E, 1209 m a.s.l., 8 October 2025, *leg*. Ștefănuț M.-M., Moroșanu A.-M., Ștefănuț S., *det.* Ștefănuț M.-M., *conf*. Ștefănuț S., BUCA *B12620*, *B12629*; Poiana Vărășoaia Peatbog, Bihor County, 46.611867° N, 22.710526° E, 1292 m a.s.l., 9 October 2025, *leg*. Ștefănuț M.-M., Moroșanu A.-M., Ștefănuț S., *det.* Ștefănuț M.-M., *conf*. Ștefănuț S., BUCA *B12630*, *B12631* (Figure 16d).

**Figure 17:**
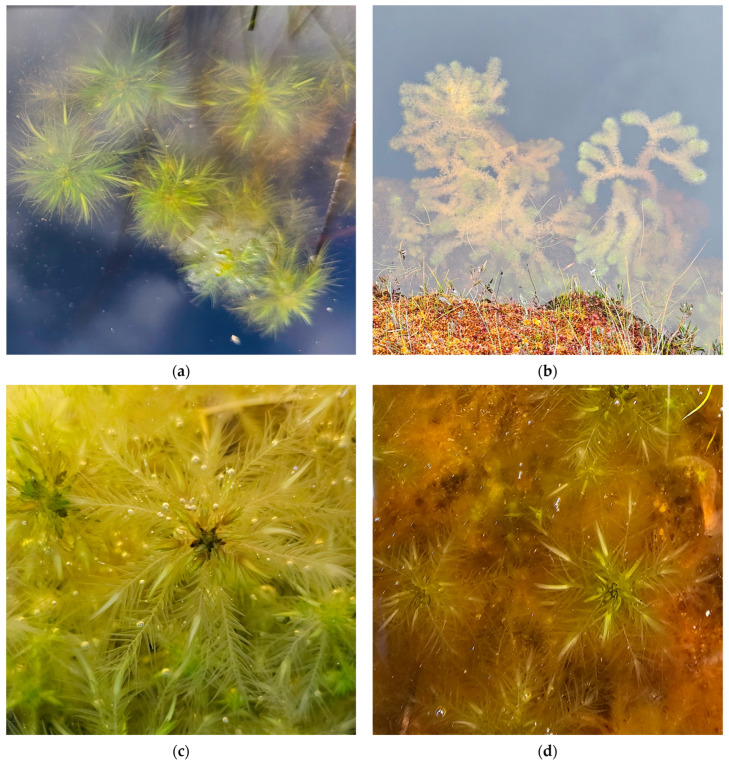
*Sphagnum cuspidatum* var. *cuspidatum*, Romania: (**a**) in the Mohoș Peatbog, Harghita County, 8 August 2025; (**b**) in Molhașul Mare de la Izbuc Peatbog, Bihor County, 8 October 2025; (**c**) in the Şesul Padiş Peatbogs, Bihor County, 9 October 2025; (**d**) in the Bălileasa Peatbog, Bihor County, 9 October 2025.

*Sphagnum cuspidatum* Ehrh. ex Hoffm. and S. *majus* (Russow) C.E.O. Jensen are two species that grow in peatland water pools. We found *S. cuspidatum* var. *cuspidatum* in water pools of Mohoș Peatbog, Harghita County, 46.136026° N, 25.901523° E, 1051 m a.s.l., 8 August 2025, *leg*. & *det*. Ștefănuț M.-M., *conf*. Ștefănuț S., BUCA *B12598* (Figure 17a), in water pools of Molhașul Mare de Izbuc Peatbog, Bihor County, 46.592650° N, 22.761262° E, 1204 m a.s.l., 8 October 2025, *leg*. Ștefănuț M.-M., Moroșanu A.-M., Ștefănuț S., *det.* Ștefănuț M.-M., *conf*. Ștefănuț S., BUCA *B12644*, *B12645* (Figure 17b), in water pools of Şesul Padiş Peatbogs, Bihor County, 46.598795° N, 22.703017° E, 1233 m a.s.l., 9 October 2025, *leg*. Ștefănuț M.-M., Moroșanu A.-M., Ștefănuț S., *det.* Ștefănuț M.-M., *conf*. Ștefănuț S., BUCA *B12646* (Figure 17c), Bălileasa Peatbog, Bihor County, 46.597129° N, 22.697110° E, 1236 m a.s.l., 9 October 2025, *leg*. Ștefănuț M.-M., Moroșanu A.-M., Ștefănuț S., *det.* Ștefănuț M.-M., *conf*. Ștefănuț S., BUCA *B12647* (Figure 17d).

**Figure 18:**
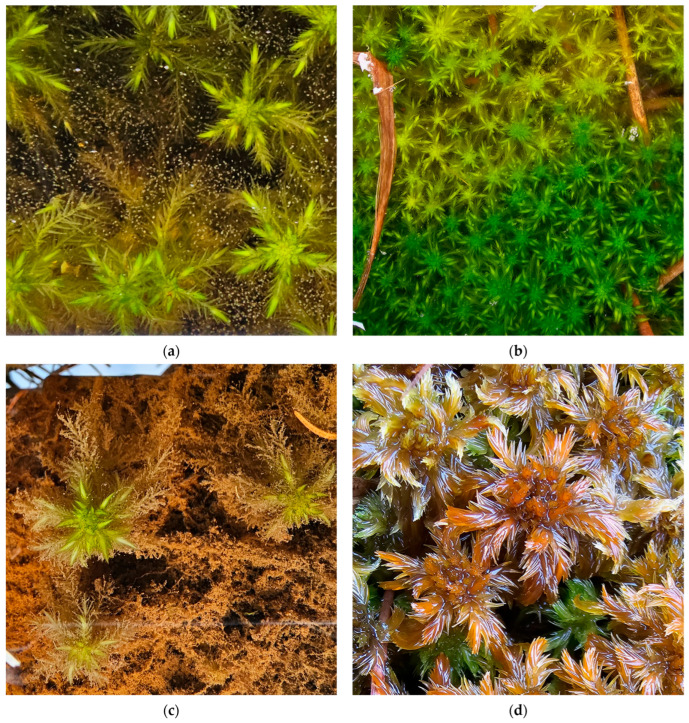
*Sphagnum majus* var. *majus*, Romania: (**a**) in the Mohoș Peatbog, Harghita County, 8 August 2025; (**b**) in Tăul fără fund din Poiana Mare—Obârșia Cloșani bog, Mehedinți County, 6 May 2025; (**c**) in the Smida Peatbog, Cluj County, 7 October 2025; (**d**) in the Molhașul Mare de la Izbuc Peatbog, Bihor County, 8 October 2025.

We found *S. majus* var. *majus* in the Hărnicești Peatbog, Maramureş County, 47.8081° N, 23.8262° E, 1007 m a.s.l., 21 April 2024, *leg*. & *det*. Ștefănuț M.M., *conf*. Ștefănuț S. & Flatberg K.I., BUCA *B12288*, TRH *126527/1* [33]; Mohoș Peatbog, Harghita County, 46.136066° N, 25.901584° E, 1051 m a.s.l., 8 August 2025, *leg*. & *det*. Ștefănuț M.-M., *conf*. Ștefănuț S., BUCA *B12592* (Figure 18a), Tăul fără fund din Poiana Mare-Obârșia Cloșani bog, Mehedinți County, 45.048260° N, 22.663480° E, 1034 m a.s.l., 6 May 2025, *leg*. & *det*. Ștefănuț M.-M., *conf*. Ștefănuț S. (Figure 18b), Smida Peatbog, Cluj County, 46.644006° N, 22.871753° E, 1019 m a.s.l., 7 October 2025, *leg*. Ștefănuț M.-M., Moroșanu A.-M., Ștefănuț S., *det.* Ștefănuț M.-M., *conf*. Ștefănuț S., BUCA *B12625* (Figure 18c), and Molhașul Mare de la Izbuc Peatbog, Bihor County, 46.591614° N, 22.762533° E, 1208 m a.s.l., 8 October 2025 *leg*. Ștefănuț M.-M., Moroșanu A.-M., Ștefănuț S., *det.* Ștefănuț M.-M., *conf*. Ștefănuț S., BUCA *B12626* (Figure 18d).

**Figure 19:**
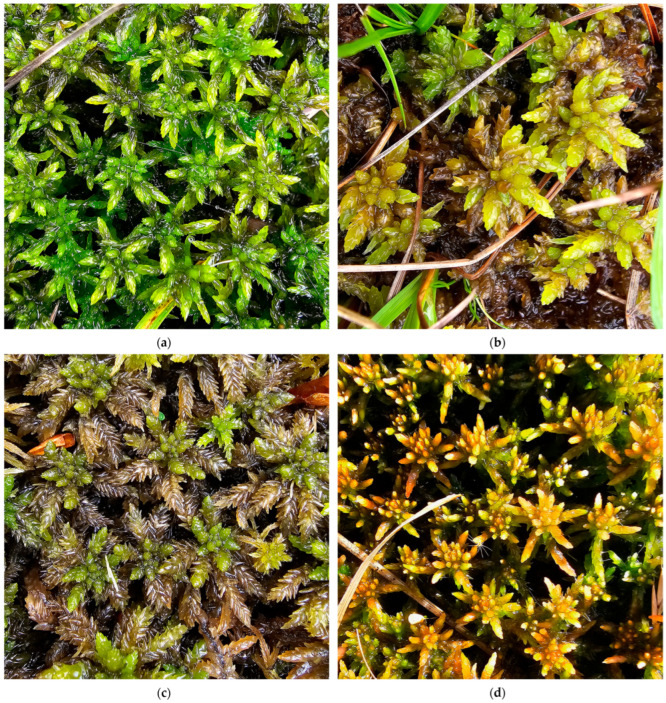
*Sphagnum inundatum* and *S. platyphyllum*, Romania: (**a**) *S. inundatum* var. *inundatum*, Vâlcelul Clinului Bog, Bucegi Massif, 12 June 2025; (**b**) *S. platyphyllum*, Vâlcelul Clinului Bog, Bucegi Massif, 12 June 2025; (**c**) *S. platyphyllum*, Mlaştina de la Goronenţi, Mehedinți County, 6 May 2025; (**d**) *S. inundatum* var. *inundatum*, Capra Valley, Făgăraș Mountains, 7 November 2024.

*Sphagnum inundatum* Russow and S. *platyphyllum* (Lindb. ex Braithw.) Warnst. are two species that usually grow together. We found both species in the subalpine water springs of the Vâlcelul Clinului Bog, Bucegi Massif, Romania, 45.356726° N, 25.462956° E, 1781 m a.s.l., 12 June 2025: *S. inundatum* var. *inundatum*, *leg*. & *det*. Ștefănuț M.-M., *conf*. Ștefănuț S., BUCA *B12500* (Figure 19a), *S. platyphyllum*, *leg*. & *det*. Ștefănuț M.-M., *conf*. Ștefănuț S., BUCA *B12530- B12532* (Figure 19b).

Moreover, we found *S. platyphyllum* from the Poiana Pelegii, Retezat Mountains, 45.34° N, 22.8932° E, 1617 m a.s.l., 21 August 2024, *leg*. Ștefănuț S., *det*. Ștefănuț M.M., *conf*. Ștefănuț S. & Flatberg K.I., BUCA *B12344*, TRH *B:126528/1* [33,44], and from Mlaştina de la Goronenţi, Mehedinți County, 44.924376° N, 22.512443° E, 814 m a.s.l., 6 May 2025, *leg*. & *det*. Ștefănuț M.-M., *conf*. Ștefănuț S. (Figure 19c).

We reported *S. inundatum* var. *inundatum* from Poiana Pelegii, Retezat Mountains, N 45.339999°, E 22.893346°, 1617 m a.s.l., 21.082024, *leg*. Ștefănuț S., *det*. Ștefănuț M-M., BUCA *B12300*, *B12301*, *B12342*, *conf*. Flatberg K.I., TRH *B:126512/1*, *B:126514/1*, and Capra Valley, Făgăraș Mountains, 45.594047° N, 24.628639° E, 1969 m a.s.l., 7 November 2024, *leg*. Ștefănuț S., *det*. Ștefănuț M-M., BUCA *B12340*, *B12341*, *conf*. Flatberg K.I., TRH *B:126513/1* (Figure 19d) [33,44]; Molhașul din Groapă Peatbog, Bihor County, Bihor Mountains, 46.594286° N, 22.761769° E, 1210 m a.s.l., 8 October 2025, *leg*. Ștefănuț M.-M., Moroșanu A.-M., Ștefănuț S., *det.* Ștefănuț M.-M., *conf*. Ștefănuț S., BUCA *B12661*.

**Figure 20:**
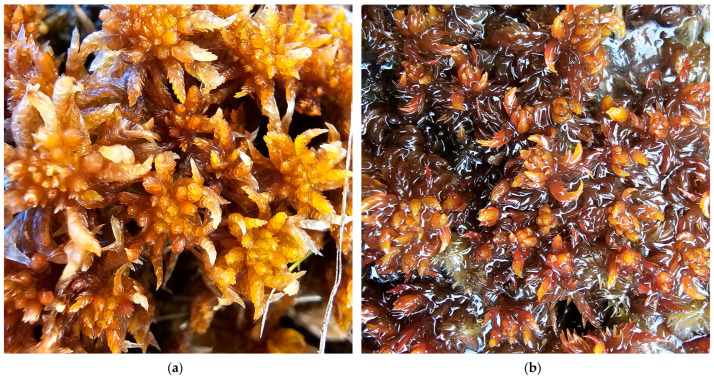
*Sphagnum inundatum* var. *gravetii* and *S. denticulatum*, Romania: (**a**) *S. inundatum* var. *gravetii*, Capra Valley, Făgăraș Mountains, 7 November 2024; (**b**) *S. denticulatum*, Molhașul din Groapă Peatbog, Bihor County, 8 October 2025.

We found *S. inundatum* var. *gravetii* (Russow) Hassel & Flatberg [45] in Capra Valley, Făgăraș Mountains, 45.594047° N, 24.628639° E, 1968 m a.s.l., 7 November 2024, *leg*. Ștefănuț S., *det*. Ștefănuț M-M., BUCA *B12338* & Flatberg K.I., TRH *B:126515/1* [33] (Figure 20a).

*Sphagnum denticulatum* Brid. and *S. inundatum* are two morphologically similar species and require the careful microscopic analysis of stem leaves [45]. We found *S. denticulatum* in the Molhașul din Groapă Peatbog, Bihor County, Bihor Mountains, 46.594345° N, 22.761824° E, 1211 m a.s.l., 8 October 2025, *leg*. Ștefănuț M.-M., Moroșanu A.-M., Ștefănuț S., *det.* Ștefănuț M.-M., *conf*. Ștefănuț S., BUCA *B12660* (Figure 20b).

### 2.5. Extinction Risk Assessment of Sphagnum Species in Romania

Using literature, herbarium, and field data, we were able to re-evaluate the *Sphagnum* species in Romania. This evaluation was carried out in accordance with the latest IUCN version of the guidelines [8].

For the conservation status assessment of the *Sphagnum* species in Romania, the extent of occurrence (EOO), area of occurrence (AOO), number of locations, habitat fragmentation, population size, conservation status of peatlands, threats and pressures, age of the reporting data, and the range of the species in Europe and the world were taken into account. The calculations of the extent of occurrence (EOO) and area of occurrence (AOO) are based on number of known localities. The rate of decline due to climatic change was not taken into account, making it the subject of a future study.

The list of *Sphagnum* species in Romania includes 35 species. Between 2021 and 2025, we recorded 35 *Sphagnum* species in the field and all have been confirmed to Romania.

The list of *Sphagnum* species in Romania and the conservation status assessment are presented in Table 1, along with comparisons with previous assessments [11,12].

## 3. Discussion

Of the 35 *Sphagnum* species with certain distribution in Romania, seven are endangered at the national level. Among the threatened species category, two species are classified as CR—Critically Endangered (5.71%); two species as EN—Endangered (5.71%); and three species as VU—Vulnerable (8.57%).

The conservation status of threatened *Sphagnum* species in Romania assessed in 2012 [11] has been refined for more than 77% of species through improved knowledge.

Our study showed that there are very limited distribution data for *Sphagnum* species in Romania, which gives the risk assessment a certain degree of uncertainty. In our study, we relied only on known localities and did not use estimates of the number of localities.

The only *Sphagnum* species with increased conservation status is *S. jensenii*, from EN (Endangered) to CR (Critically Endangered) (AOO = EOO = 8 km^2^), due to rejected reports of *S. annulatum*, which were included as synonyms of *S. jensenii*.

The conservation status of the species *Sphagnum wulfianum* remains unchanged as EN (Endangered) (AOO = 24 km^2^, EOO = 1,945.8 km^2^); only the criteria is changed from B2b(iii,iv); C1 to A1c; C1. This is because, although it has been reported from several bogs in Romania, the data are very old and the species has been found only once in the last 49 years. We have searched for this species in the peatbogs where it was previously reported (Teșna Românești, Poiana Stampei, Drăgoiasa, Valea Stânii, Grădinița, and Apa Roșie Peatbogs), but only in Apa Roșie Peatbogs have we found it.

The records of *Sphagnum wulfianum* from Apa Roșie Peatbog in Romania are at the southernmost limit of its global range.

The conservation status of *Sphagnum papillosum* was changed from CR B1ab(ii,iii) + B2ab(ii,iii) to EN B2ab(ii,iii,iv) in 2025 [20], but, with the new records from Bucegi Massif and Bihor Mountains, the area of occupancy (AOO = 24 km^2^) and the extent of occurrence (EOO = 26,643.6 km^2^) are larger. For these reasons, the conservation status of *S. papillosum* in Romania is now VU B2ab(ii,iii,iv). We believe that the species is much more widespread than currently known and further research could lead to its assessment as NT (Near Threatened) or perhaps even LC (Least Concern). A similar situation applies to *Sphagnum balticum* and *S. obtusum*, which, following improved field research, may no longer be included in the threatened categories.

Until now, *Sphagnum affine* had not been evaluated (NE) because of a lack of data [11,12], but we confirm the presence of this species in one site in Romania (AOO = EOO = 4 km^2^), and the conservation status is changed to CR B1ab(ii,iii) + B2ab(ii,iii). In the field, *S. affine* is very difficult to distinguish from *S. palustre*, especially since the two species grow together. On this basis, we suggest that *S. affine* is much more widespread in Romania, but it has been overlooked.

With two new records of *Sphagnum subfulvum* subsp. *subfulvum* to Romania (AOO = 12 km^2^, EOO = 18,344.9 km^2^), the conservation status of this species has been changed from CR B1ab(ii,iii) + B2ab(ii,iii) [38] to EN B2ab(ii,iii,iv).

New records of *Sphagnum balticum* (AOO = 32 km^2^, EOO = 31,923.2 km^2^) and *S. obtusum* (AOO = 24 km^2^, EOO = 35,773.9 km^2^) in Romania have led to the change in the conservation status of these species from EN B2ab(ii,iii,iv) to VU B2ab(ii,iii,iv).

New distribution data for the species *Sphagnum denticulatum*, *S. majus*, and *S. inundatum* have led to their classification in the NT (Near Threatened) category from VU (Vulnerable) and from EN (Endangered) for *S. tenellum*.

Because *Sphagnum cuspidatum* and *S. majus* grow in water pools and the peatlands with water pools in Romania represent less than 15% of all *Sphagnum* peatlands in Romania, thar conservation status of these species is NT—Near Threatened. *Sphagnum majus* was assessed in 2012 as a VU (Vulnerable) species, but our research proved that it was confused with *S. cuspidatum* and is much more frequent than has been reported. For example, in the Mohoș Peatbog, Harghita County, where we found both species in 2025, only *S. cuspidatum* was reported previously.

The less common species with an extent of occurrence (EOO) > 20,000 km^2^, an area of occurrence (AOO) > 2000 km^2^, or a number of locations between 11 and 20, such as *Sphagnum cuspidatum*, *S. denticulatum*, *S. inundatum*, *S. fimbriatum*, *S. majus*, *S. platyphyllum S. riparium*, and *S. tenellum*, were assessed as NT—Near Threatened.

The most common *Sphagnum* species in Romania is *S. girgensohnii*, which is found in almost all oligotrophic peatbogs, mountain forests and subalpine areas, from 600 to 2500 m a.s.l. Almost as common is *S. squarrosum*, found in Romania on the edges of lakes, peatbogs or mountain streams.

The frequently found species with an extent of occurrence (EOO) > 30,000 km^2^, an area of occurrence (AOO) > 3000 km^2^, or occurrence in more than 20 locations in Romania, such as *Sphagnum angustifolium*, *S. capillifolium*, *S. centrale*, *S. compactum*, *S. contortum*, *S. divinum*, *S. fallax*, *S. flexuosum*, *S. fuscum*, *S. girgensohnii*, *S. medium*, *S. palustre*, *S. quinquefarium*, *S. rubellum*, *S. russowii*, *S. squarrosum*, *S. subnitens*, *S. subsecundum*, *S. teres*, and *S. warnstorfii*, were assessed as LC—Least Concern.

The habitats richest in *Sphagnum* species are the peatbogs with water pools, which have over 15 species, while the peatbogs without water pools have fewer species, because they lack species that are more dependent on water, such as *S. cuspidatum*, *S. denticulatum*, *S. majus*, *S. balticum*, *S. tenellum*, *S. jensenii*, *S. contortum*, *S. inundatum*, *S. subsecundum*, *S. platyphyllum*, and *S. teres.* Another habitat with a high diversity of *Sphagnum* species is represented by permanent subalpine springs, where in addition to the above species, there are also *S. compactum*, *S. papillosum*, and *S. subfulvum*.

The peatland habitats already afforested with pine (*Pinus sylvestris* L.) seem to be losing the great diversity of *Sphagnum* species they once had, being now dominated by common species such as *S. capillifolium*, *S. divinum*, *S. girgensohnii*, *S. centrale*, *S. medium*, *S. palustre*, and *S. squarrosum*; sometimes with *S. fuscum*, *S. rubellum*, *S. russowii*, and *S. subnitens*; and less often with *S. warnstorfii.* If these habitats are still well moistened, the species that prefer hollows appear, such as *S. angustifolium*, *S. fallax*, and *S. flexuosum*.

As a result, habitats that still have water pools are a priority for protection and conservation; restoration measures should focus on preserving/restoring water pools and blocking drainage channels.

Between 1950 and 1990, the peatbogs in Romania were extensively drained to reclaim land for agriculture. The drainage channels are still active in many peatlands, but some of them have been blocked during restoration projects carried out in Romania over the last 10 years, in over 45 peatlands. Positive effects, including a water table rise exceeding 50 cm in some peatlands, were observed within a year, facilitating the expansion of hollow-dwelling species such as *S. fallax*, *S. angustifolium*, *S. flexuosum*, *S. contortum*, and *S. subsecundum*.

Even though *Sphagnum* species in Romania are included in Annex V of the European Habitats Directive and do not require legal strict protection, we consider the species assessed here to be threatened: *Sphagnum affine*, *S. balticum*, *S. jensenii*, *S. obtusum*, *S. papillosum*, *S. subfulvum*, and *S. wulfianum* should be included on the list of nationally protected species. Among them, *Sphagnum affine*, *S. jensenii*, *S. subfulvum*, and *S. wulfianum* are priority species.

## 4. Materials and Methods

The distribution of *Sphagnum* species in Romania was compiled using literature, herbaria, open access databases [33] and field data during the period 2001–2025, especially 2021–2025.

More than 100 peatlands across Romania were investigated:

- Maramureș County: Vlășchinescu Peatbog, Hărnicești Peatbog, Tăurile Chendroaiei (Tăul de la Gutâi) Peatbog;
- Suceava County: Lucina-Găina Peatbog, Tinovul de lângă drum-Lucina Peatbog, Lucina Est Peatbog, Lucina Vest Peatbog, Tinovul Sângerozanei Peatbog; Tinovul cel mare de la Coșna Peatbog, Tinovul Jinului Peatbog, Teșna Românești Peatbog, Teșna Peatbog, Bahnele Bancului I Peatbog, Bahnele Bancului II Peatbog, Poiana Stampei Peatbog, Pilugani Peatbog, Șaru Dornei Peatbog, Drăgoiasa Peatbog;
- Bistrița-Năsăud County: Grădinița Peatbog, Larion Peatbog;
- Călimani Mountains: Iezer Lake Peatbogs;
- Harghita County: Mohoș Peatbog, Sâncrăieni-Sântimbru Peatbogs, Dumbrava Harghitei Peatbog;
- Covasna County: Apa Roșie Peatbog, Apa Lină Peatbog, Comandău Peatbog;
- Siriu Mountain: Lacul cu ochi Peatbog;
- Buzău County: Manta Lake Peatbog;
- Prahova County: Bâlbâitoarea Peatbog;
- Bucegi Mountains: Nucet-Blana Peatbogs, Lăptici Peatbog—Blana Valley, Lăptici II—Scândurilor Valley Peatbog, the peatbogs of Lăptici Valley, Horoaba Valley Peatbog;
- Ierzer-Păpușa Mountains: Iezer Lake Peatbogs;
- Sibiu County: Mlaca Tătarilor Peatbog, Lacul lui Vizante Peatbog, Avrig Peatbogs;
- Făgăraș Mountains: Zârna Lake Peatbogs, Valea Rea Peatbogs; Podragu Lake Peatbogs, Capra Lake Peatbogs, Bâlea Lake Peatbogs, Puha Valley Peatbogs;
- Vâlcea County: Mosoroasa Peatbog;
- Șureanu Mountain: Iezerul Șureanu Peatbog;
- Parâng Mountains: Câlcescu (Gâlcescu) Peatbog, Gaura Valley Peatbogs;
- Alba County: Mluha Peatbog; Tăul fără fund de la Băgău Peatbog;
- Hunedoara County: Peșteana Peatbog;
- Retezat Mountains: Bucura Lake Peatbogs, Lia Lake Peatbogs, Galeș Lake Peatbogs; Tăul dintre Brazi Peatbog; Poiana Pelegii Peatbog;
- Țarcu Mountains: Mătania Valley Peatbogs;
- Mehedinți County: Tăul fără fund de la Poiana Mare-Obârșia Peatbog; Mlaștina în trepte din Poiana Mare Obârșia Cloșani Peatbog; Tăul fără fund de la Busești, Gornenți Peatbog;
- Gilău Mountains: Molhașul de la Călățele Peatbog; Dâmbu Negru-Între Drumuri Peatbog; Negrușul Finciului-Rovina Mare Peatbog; Molhașul de la Râșca—Dealul Negru Peatbog; Dâmbu Negru-La Pod Peatbog;
- Cluj County: Tinovul Smida; Între Șimone Peatbog;
- Bihor Mountains: Molhașurile din Valea Izbucelor; Molhașul din Groapă; Seria de mlaștini de la Sâvla; Șesul Padiș Peatbogs; Bălileasa Peatbog; Tăul fără fund Peatbog; Poiana Vărășoaia Peatbogs;
- Bihor County: Tinovul de la Ic Ponor; Călineasa-Izbuc Peatbog.

**Figure 21:**
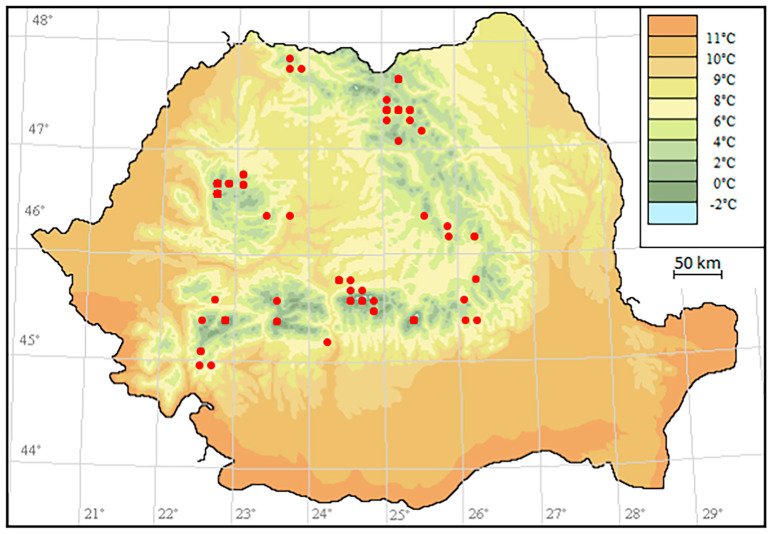
Mean annual temperature map, Romania: the investigated peatlands (red dots).

The RoBioAtlas (2023) web application [46] was used to create the map of the investigated peatlands (Figure 21).

The field data were collected for each occurrence: geographic coordinates (decimal degrees) and elevation using a GPS receiver. A review of scientific publications was conducted, and all distribution data were registered in a database. When coordinates were not available, locations were georeferenced using Google Earth Pro, Google Maps, and military survey maps for Romania with an assumed error of less than 1 km. The extent of occurrence (EOO) and the area of occurrence (AOO) were calculated in ArcGIS 10.7.1 using a 2 km square grid [47].

The conservation assessment was carried out according to the Guidelines for Using the IUCN Red List Categories and Criteria, Version 16 [8]. The *Sphagnum* taxa in Romania was assessed at the species level. The *Sphagnum* species, which, in previous assessments [11,12], were included in the categories DD—Data Deficient (three species) and NE—Not Evaluated (one species) were re-evaluated in this study.

In assessing the conservation status of *Sphagnum* species, the following factors were also taken into account: peatlands fragmentation in Romania [21], current threats to peatlands, including climate change [48], and *Sphagnum* species’ distribution in Romania.

For the *Sphagnum* species distribution maps, the methodology developed in *The Liverwort and Hornwort Atlas of Romania* was used [49] (pp. 13–19); it uses grid maps to mark the grid cells in which the species was reported at least once [50] (p. 34). The reference grid used is the GEOCOD (Geographic Coordinates Code), but the UTMG (Universal Transverse Mercator Grid) can be used too. From 2023, the RoBioAtlas web application was developed and is available online with free access [46]. The Web Application uses GPS Coordinates (WGS 84) or UTMG (Universal Transverse Mercator Grid) to generate species, habitats, and ecosystem distribution maps for Romania.

The *Sphagnum* species nomenclature used is according to Hodgetts et al. (2020) [51] and Hassel et al. (2025) [45].

The RoBioMeasurements (2025) web application [52] was used to insert the scale in the pictures.

No generative artificial intelligence (GenAI) was used in preparing this study.

## 5. Conclusions

The new distribution data show that the assessment of the conservation status of *Sphagnum* species in Romania is constantly changing. Barriers include the existing information gap due to the limited number of specialists capable of accurately identifying *Sphagnum* species, the lack of published studies, the extensive work still required, and the large number of peatbogs (over 250) that should be reassessed at the national level.

We hope that, by the end of the doctoral research period, in 2028, a clearer picture of the distribution of *Sphagnum* species in Romania will emerge, along with improved assessment of them from a conservation point of view. Additionally, we anticipate that new species may be reported in Romania.

From this temporal perspective, future research efforts will focus on the correlation of *Sphagnum* species’ presence with the stability and variability of the environment over short- and medium-term periods. We will assess these factors through comprehensive phytodynamic analyses, aiming to capture the potential transitions between different types of wetlands—reflecting stable, secondary, and transitional conditions—based on species presence, distribution, abundance, and spontaneous dynamics. This approach attempts to improve ecological understanding and guide more adaptive and effective management of the conservation of *Sphagnum*-dominated peatland ecosystems under permanently changing environmental conditions.

The conservation assessment of *Sphagnum* species in Romania carried out here represents a reference for future research dedicated to their distribution, conservation status, and ecology.

We expect that, after three years of research, the conservation status of *Sphagnum* species in Romania will be improved by more than 40% and at least three species will no longer be on the endangered species list, not due to changes in range or quality habitats, but due to improved knowledge.

## Figures and Tables

**Table 1:** Checklist and conservation status (Red List categories) of *Sphagnum* species in Romania.

Taxon	Ștefănuț and Goia, 2012 [11]	Hodgetts and Lockhart, 2020 [12]	This Study (2025)
Subgenus *Rigida* (Lindb.) A. Eddy			
*Sphagnum compactum* Lam. & DC.	LC	LC	LC
Subgenus *Sphagnum*			
*Sphagnum affine* Renauld & Cardot	NE	NE	**CR** B1ab(ii,iii) + B2ab(ii,iii)
*Sphagnum centrale* C.E.O. Jensen	LC	LC	LC
*Sphagnum divinum* Flatberg & Hassel	-	-	LC
* *Sphagnum magellanicum* Brid.	LC	-	-
*Sphagnum medium* Limpr.	-	-	LC
*Sphagnum palustre* L.	LC	LC	LC
*Sphagnum papillosum* Lindb.	**CR** B1ab(ii,iii) + B2ab(ii,iii)	**CR**	**VU** B2ab(ii,iii,iv)
Subgenus *Acutifolia* (Russow) A.J. Shaw			
Section *Squarrosa* (Russow) Schimp.			
*Sphagnum squarrosum* Crome	LC	LC	LC
*Sphagnum teres* (Schimp.) Ångstr.	LC	LC	LC
Section *Polyclada* (C.E.O.Jensen) Horrell			
*Sphagnum wulfianum* Girg.	**EN** B2b(iii,iv); C1	**EN**	**EN** A1c; C1
Section *Acutifolia* Wilson			
*Sphagnum capillifolium* (Ehrh.) Hedw.	LC	LC	LC
*Sphagnum fimbriatum* Wilson	LC	LC	NT
*Sphagnum fuscum* (Schimp.) H. Klinggr.	LC	LC	LC
*Sphagnum girgensohnii* Russow	LC	LC	LC
* *Sphagnum molle* Sull.	DD	DD	-
*Sphagnum quinquefarium* (Braithw.) Warnst.	LC	LC	LC
*Sphagnum rubellum* Wilson	LC	LC	LC
*Sphagnum russowii* Warnst.	LC	LC	LC
*Sphagnum subfulvum* Sjörs	-	-	**EN** B2ab(ii,iii,iv)
*Sphagnum subnitens Russow & Warnst*.	LC	LC	LC
*Sphagnum warnstorfii* Russow	LC	LC	LC
Subgenus *Subsecunda* (Lindb.) A.J. Shaw			
*Sphagnum contortum* Schultz	LC	LC	LC
*Sphagnum denticulatum* Brid.	**VU** B2ab(ii,iii,iv)	**VU**	NT
*Sphagnum inundatum* Russow	**VU** B2ab(ii,iii,iv)	**VU**	NT
*Sphagnum platyphyllum* (Lindb. ex Braithw.) Warnst.	NT	NT	NT
* *Sphagnum pylaesii* Brid.	-	-	-
*Sphagnum subsecundum* Nees	NT	NT	LC
Subgenus *Cuspidata* Lindb.			
*Sphagnum angustifolium* (C.E.O. Jensen ex Russow) C.E.O. Jensen	LC	LC	LC
* *Sphagnum annulatum* H. Lindb. ex Warnst.	DD	DD	-
*Sphagnum balticum* (Russow) C.E.O. Jensen	**EN** B2ab(ii,iii,iv)	**EN**	**VU** B2ab(ii,iii,iv)
*Sphagnum cuspidatum* Ehrh. ex Hoffm.	LC	LC	NT
*Sphagnum fallax* (H. Klinggr.) H. Klinggr.	LC	LC	LC
*Sphagnum flexuosum Dozy & Molk*.	LC	LC	LC
*Sphagnum jensenii* H. Lindb.	**EN** B2ab(ii,iii,iv)	**EN**	**CR** B2ab(ii,iii,iv)
*Sphagnum majus* (Russow) C.E.O. Jensen	**VU** B2ab(ii,iii,iv)	**VU**	NT
*Sphagnum obtusum* Warnst.	**EN** B2ab(ii,iii,iv)	**EN**	**VU** B2ab(ii,iii,iv)
* *Sphagnum pulchrum* (Lindb. ex Braithw.) Warnst.	DD	DD	-
*Sphagnum riparium* Ångstr.	LC	LC	NT
*Sphagnum tenellum* (Brid.) Pers. ex Brid.	**EN** B2ab(ii,iii,iv)	**EN**	NT

* Excluded species; CR—Critically Endangered; EN—Endangered; VU—Vulnerable; NT—Near Threatened; LC—Least Concern; DD—Data Deficient; NE—Not Evaluated; B1—Extent of occurrence (EOO); B2—Area of occurrence (AOO); a—Severely fragmented OR Number of locations; b—Continuing decline observed, estimated, inferred or projected in any of: (ii) area of occupancy, (iii) area, extent and/or quality of habitat, (iv) number of locations or subpopulations; A1c—Population reduction suspected for a decline in area of occupancy (AOO), extent of occurrence (EOO) and/or habitat quality; C1—An observed, estimated or projected continuing decline.

## Data Availability

The data presented in this study are either available in the article, or available on request from the authors.

## Abbreviations

The following abbreviations are used in this manuscript:

BP	Hungarian National Museum Public Collection Centre, Budapest, Hungarian Natural History Museum, Hungary; Botanical Department, Herbarium BP, Bryophyte herbarium, *S*—*Sphagnum* collection
BRNU	Masaryk University, Faculty of Science, Department of Botany and Zoology, Brno, Czech Republic; Herbarium BRNU
BUCA	Romanian Academy Herbarium, Institute of Biology Bucharest, Romania; Herbarium BUCA, *B*—Bryophyte herbarium
CL	Herbarium of Babeș-Bolyai University in Cluj-Napoca, Romania; Herbarium CL
EGR	Herbarium of Eszterházy Károly Catholic University in Eger, Hungary; Herbarium EGR
EU	European Union
FRE	Flora Romaniae Exssicata
IUCN	The International Union for Conservation of Nature
TRH	Trondheim Herbarium, Norwegian University of Science and Technology Museum, Norway; Herbarium TRH, *B*—Bryophyte herbarium
